# Retained Placenta Percreta with Acquired Uterine Arteriovenous Malformation—Case Report and Short Review of the Literature

**DOI:** 10.3390/diagnostics12040904

**Published:** 2022-04-05

**Authors:** Tudor Butureanu, Raluca Anca Balan, Razvan Socolov, Nicolae Ioanid, Demetra Socolov, Dumitru Gafitanu

**Affiliations:** 1 Department of Mother and Child Medicine, Grigore T. Popa University of Medicine and Pharmacy, 16 Universitatii Str., 700115 Iaşi, Romania; tudorandreib@gmail.com (T.B.); socolov.razvan@gmail.com (R.S.); demetrasocolov@gmail.com (D.S.); dumitru.gafitanu@umfiasi.ro (D.G.); 2 Department of Morphofunctional Sciences I-Histology, Grigore T. Popa University of Medicine and Pharmacy, 16 Universitatii Str., 700115 Iaşi, Romania; 3 Department of Gynecologic Oncology, Regional Institute of Oncology, 2-4 G-ral Berthelot Str., 700483 Iaşi, Romania

**Keywords:** placenta accreta spectrum (PAS), uterine arteriovenous malformation (AVM), retained product of conception (RPC), uterine hemorrhage, Doppler ultrasound

## Abstract

Placenta accreta spectrum disorder (PAS) has an increased frequency due to the high number of cesarean sections. The abnormal placentation associated with a retained placenta can cause persistent uterine bleeding, with ultrasound Doppler examination being the main choice to assess the uterine hemorrhage. An acquired uterine arteriovenous malformation (AVM) may occur because of uterine trauma, spontaneous abortion, dilation and curettage, endometrial carcinoma or gestational trophoblastic disease. The treatment for abnormal placentation associated with AVM can be conservative, represented by methotrexate therapy, arterial embolization, uterine curettage, hysteroscopic loop resection or radical, which takes into consideration total hysterectomy. Therapeutic management always considers the degree of placental invasion, the patient hemodynamic state and fertility preservation. Considering the aspects described, we present a case of retained placenta percreta associated with acquired uterine AVM, with imagistic and clinical features suggestive of a gestational trophoblastic disease, successfully treated by hysterectomy, along with a small review of the literature, as only a few publications have reported a similar association of diagnostics and therapy.

## 1. Introduction

Placenta percreta, part of the placenta accreta spectrum disorder (PAS) or abnormally invasive placenta (AIP), represents a form of invasive placentation, a condition defined by a firm placental adhesion or invasion in the uterine wall, chorionic villi directly adhering to the muscular uterine wall, without the presence of decidua basalis or deep invasion of the myometrium [1,2,3]. Thus, the placenta is no longer able to detach spontaneously after delivery, leading to serious complications, such as massive life-threatening maternal hemorrhage, which often requires a hysterectomy surgical approach [1,3,4,5,6].

PAS has an increasing incidence, most likely due to the increase in the number of cesarean sections, which represents a major risk factor in the development of subsequent abnormal placentation [3,7].

The ultrasound diagnosis, with combined grayscale and color Doppler images, is the main choice for the assessment of antepartum or postpartum hemorrhage due to abnormal placentation or retained placenta [1,8]. Early diagnosis is crucial in order to prevent short-term and long-term maternal complications [1,9].

Uterine arteriovenous malformation (AVM), a rare condition, but frequently undiagnosed, can be congenital or acquired [10,11,12,13]. The etiology of acquired AVM comprises uterine trauma [10,14], spontaneous abortion [10,15], dilation and curettage [10,15,16], endometrial carcinoma [17] or gestational trophoblastic disease [10,18].

The therapeutic strategy for abnormal placentation includes conservative or surgical management. Conservative management with the uterus and placenta left in situ involves methotrexate, as well as arterial embolization, curettage and hysteroscopic loop resection, while the surgical approach mainly takes into consideration hysterectomy [4,6,19]. The therapeutic choice considers the degree of placental invasion or other maternal medical contexts, such as infections, a hemodynamic state or preserving fertility [4]. 

The association of retained placenta or product of conception with PAS may also need surgery [20].

Given the aspects mentioned above, we present a case of retained placenta percreta associated with acquired uterine AVM mimicking a gestational trophoblastic disease, surgically treated by hysterectomy, with a very good outcome, along with a short review of the literature, considering that few publications have reported a similar association of diseases [20,21,22,23,24].

## 2. Case Presentation

A 29-year-old woman with three previous births presented into the emergency room of the Gynecological Department with heavy bleeding and the absence of menstruation for 14 weeks. The COVID-19 test was negative. The patient’s history revealed three previous cesarean sections, without any other pathological medical history.

It is worth mentioning that the patient was previously admitted to another Gynecological Department for the same symptomatology when β-HGC was 160 mUI/mL, and the histopathological diagnosis was consistent with an incomplete abortion.

**Figure 1:**
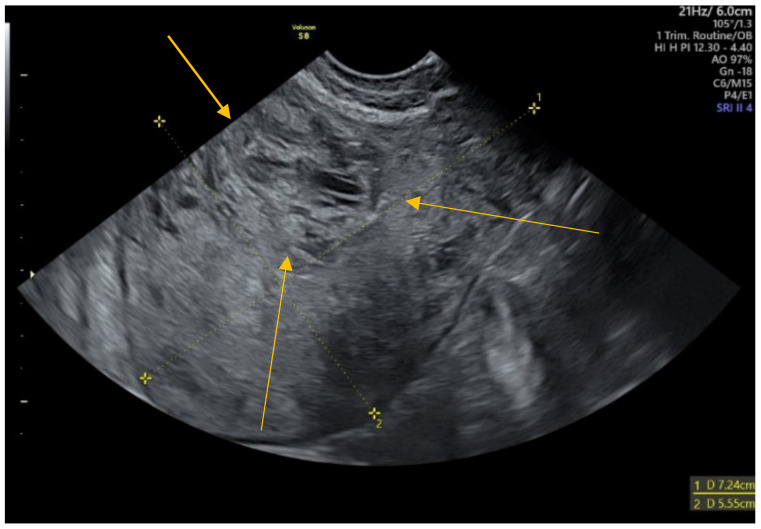
Abnormal echostructure of the uterus anterior wall.

The ultrasound transvaginal examination performed in our clinic described a 72/55 mm uterus, with an inhomogeneous structure. A heterogeneous area of 30/24 mm, with a color Doppler signal in the entire thickness of the anterior wall, extended into the endometrial cavity (Figure 1).

**Figure 2:**
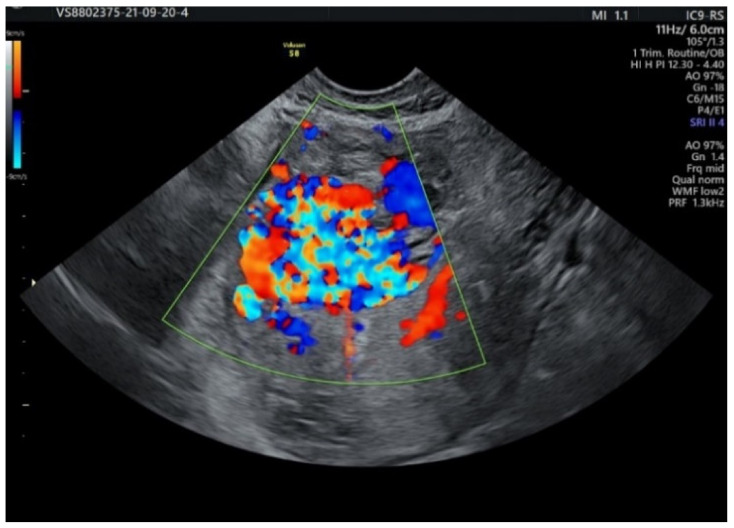
Uterine vascularization with mixed vascular Doppler signal.

The image raised suspicion of arteriovenous malformation, with a multicystic appearance and intense vascularization in the color Doppler flow. There was no echogenic chorionic ring, a specific ultrasound marker of the gestational sac. The adnexa presented a normal sonographic appearance, with suspicion of postoperative adhesions, because of the reduced and painful mobility of the left ovary. The diagnosis was improved with the help of the Doppler ultrasound system, which showed increased vascularization with different flow speeds, arterial and venous (Figure 2).

**Figure 3:**
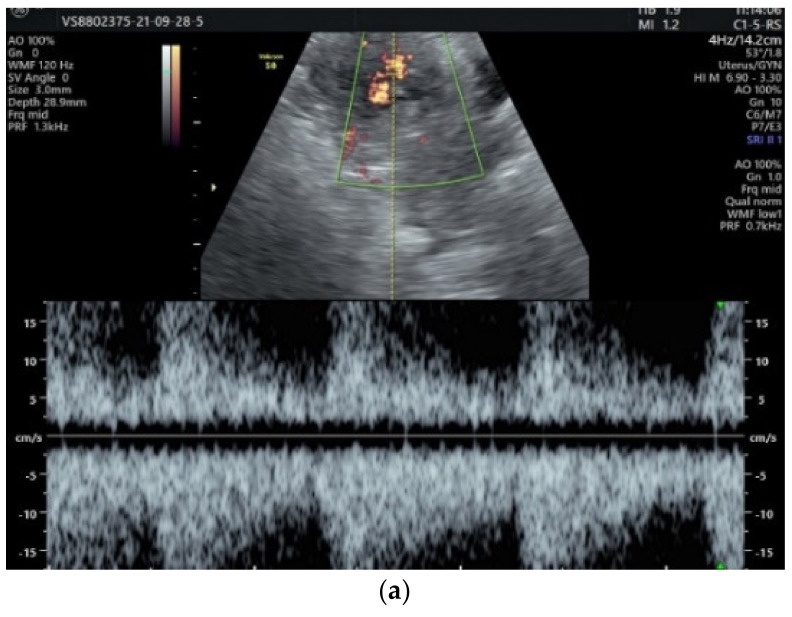

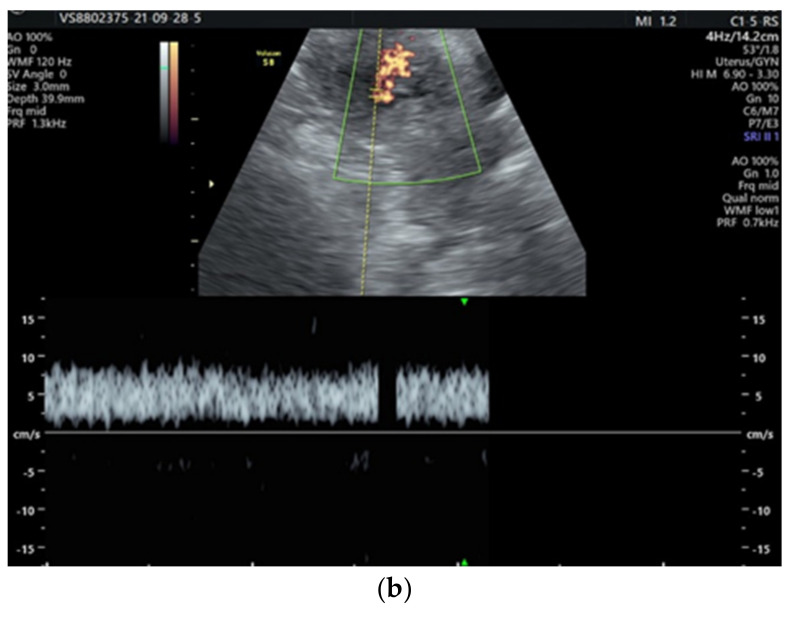
Pulsatile Doppler ultrasound: (**a**) Arterial flow; (**b**) venous flow.

Pulsatile Doppler specified the hemodynamic characteristics of the arteriovenous shunt, as well as the alternation between predominantly systolic arterial flow and venous diastolic flow (Figure 3a,b).

At admission, hemoglobin (Hb) was 6 g/dL. Because the iron therapy only slightly increased the hemoglobin levels at 8 g/dL, after decreasing again to 6.7 g/dL, the patient was referred for transfusion in order to perform surgery. In our clinic, the first β-HGC value was 40 mUI/mL, with no other modified biochemical and hematological parameters.

Because, during hospitalization, the gonadotrophin value continued to increase to 427 mUI/mL, a 48 mg methotrexate treatment was instituted. Subsequently, β-HGC decreased to 90 mUI/mL, and a second course of methotrexate of 40 mg was administered.

**Figure 4:**
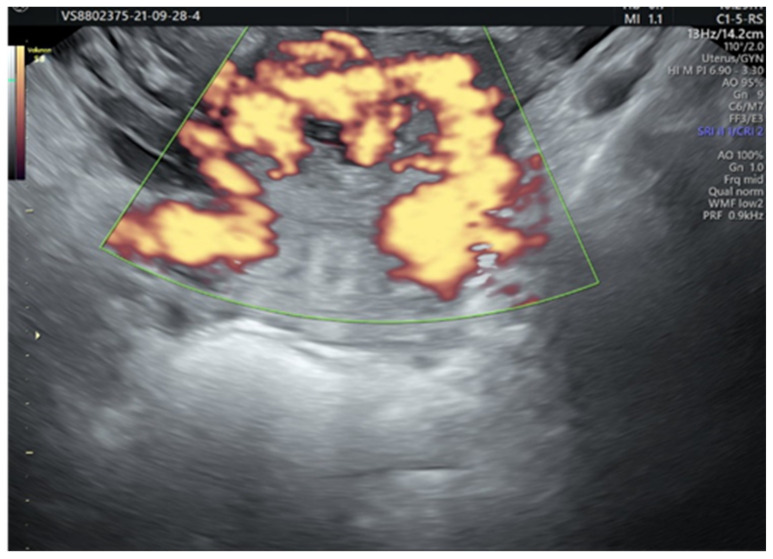
Intensely vascularized area; communication with the uterine vascularization.

The patient underwent a second ultrasound examination, which revealed the persistence of the intensely vascularized area from the anterior uterine wall (Figure 4).

The absence of the gestational sac, the moderate values of β-HGC and the persistence of the highly vascularized area in the anterior uterine wall at ultrasound examination represented the reasons why she was referred to an abdomino-pelvic MRI examination, which described an expansive heterogeneous lesion, with numerous tubular vascular structures and indistinct borders, measuring 51/48/48 mm. The lesion distorted the junctional layer and protruded into the uterine cavity, creating a mass effect. The images were suggestive of a hypervascularized variant of gestational trophoblastic disease, raising suspicion of a placental trophoblastic tumor or choriocarcinoma.

The biopsy curettage performed in our clinic revealed only a few cells with suspected atypical morphology, embedded in massive blood clots.

New ultrasonography was performed, which revealed a uterus of 86/50 mm, with a heterogeneous structure, and the same inhomogeneous area of 31/32 mm on the anterior wall, with increased arteriovenous vascularization, maintaining the suspicion of intrauterine AVM. The adnexa had also a normal ultrasound aspect.

Considering the MRI suspicion of diagnosis and the persistent bleeding, a multidisciplinary team composed of gynecologists, an oncologist, and anesthesiologists decided to perform a total hysterectomy. The surgical procedure involved a total hysterectomy, bilateral salpingectomy, left oophorectomy, with right external iliac lymph node sampling and extended adhesiolysis. The post-operative evolution was uneventful, with the patient requiring only a new transfusion.

**Figure 5:**
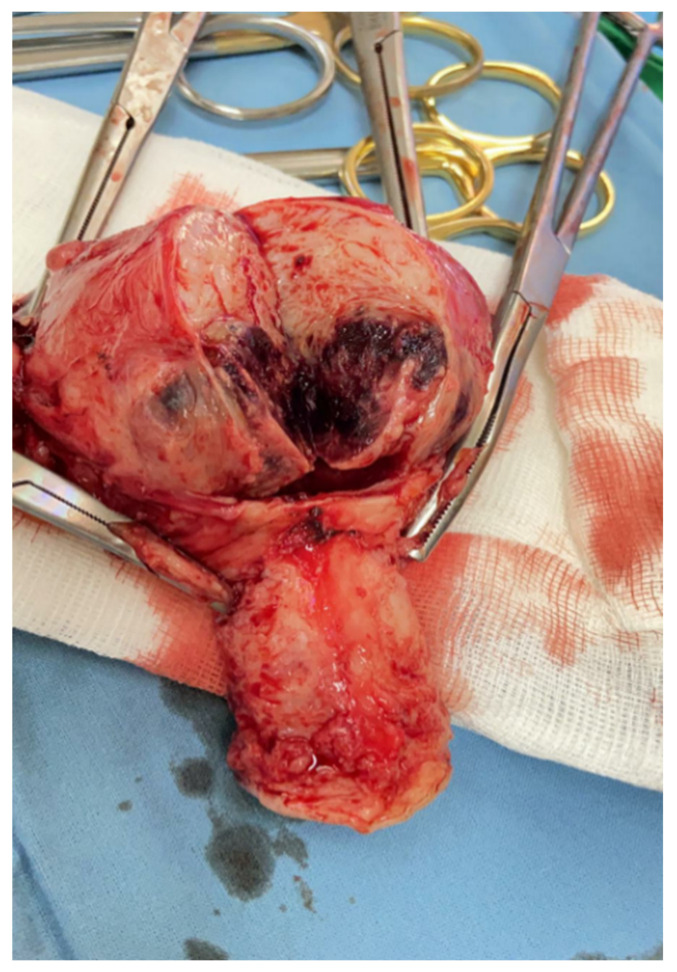
Placenta percreta with abnormal vascularization reaching the lower uterine segment.

**Figure 6:**
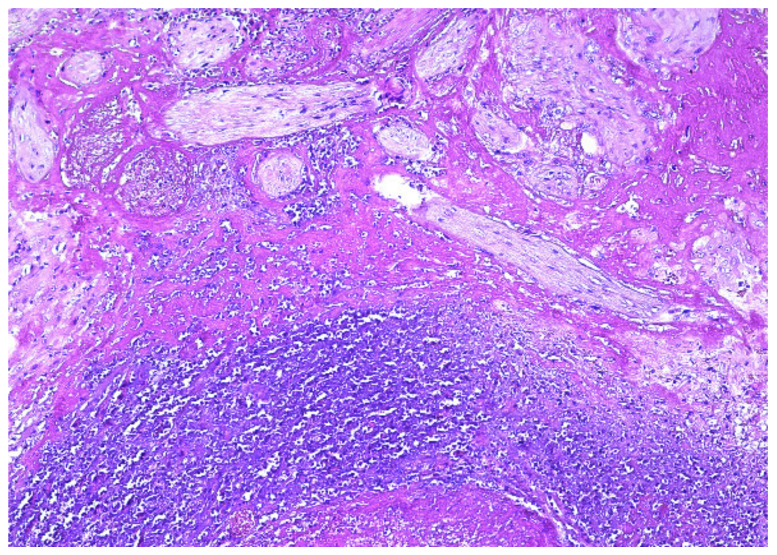
Fibrotic chorionic villi infiltrating myometrium (HE × 10).

**Figure 7:**
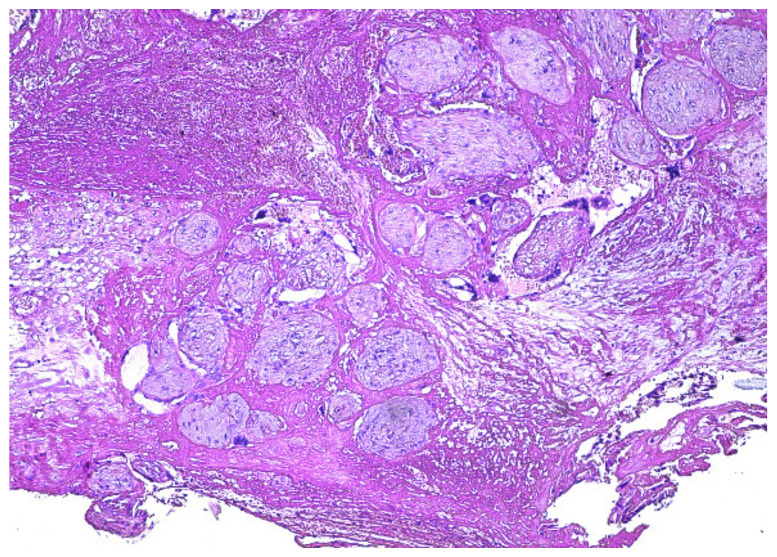
Marked fibrosis of chorionic villi, which invade the full thickness of the uterine wall (HE × 10).

**Figure 8:**
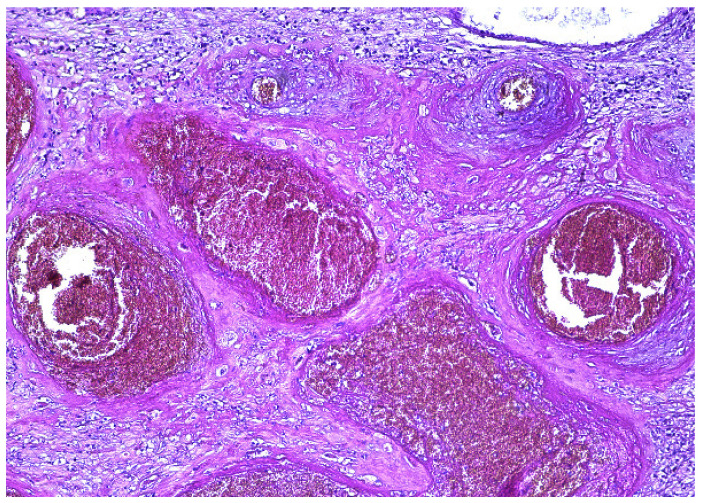
Dilated irregularly shaped blood vessels and thrombosis (HE × 10).

**Figure 9:**
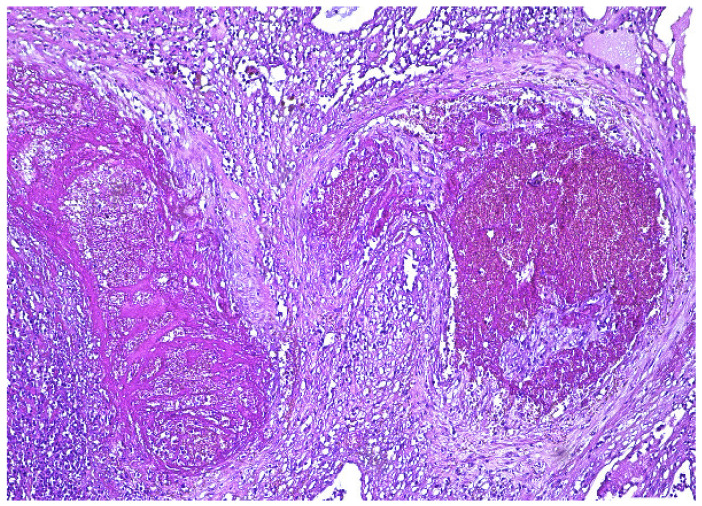
Vascular malformation and thrombi (HE × 10).

The surgical specimen was submitted to the Pathology Department, where it was grossly (Figure 5) and microscopically examined (Figure 6, Figure 7, Figure 8 and Figure 9).

The retained placenta percreta was documented by histologic examination, through extensive sampling, which revealed, in the endometrium and myometrium, large areas of necrosis and hemorrhage, focal microcalcifications, diffuse mixed inflammatory infiltrate, a few areas of decidua and bundles of predominantly necrotic muscle fibers, infiltrated by fibrotic and degenerating chorionic villi, sometimes lined by a discontinuous trophoblast involving the entire uterine wall (Figure 6 and Figure 7). Arteriovenous malformation was also histologically documented by numerous myometrial irregularly shaped and dilated blood vessels, containing well-organized thrombi, with a highly branched fibrin network and abnormal communication between arteries and veins (Figure 8 and Figure 9). These aspects intersected the entire uterine wall, toward the isthmus, where the glandular epithelium presented syncytial and hobnail-type reactive changes. Morphological aspects compatible with histiocytic mixed follicular hyperplasia in the right external iliac lymph node completed the diagnosis.

## 3. Discussion

In the placenta accreta spectrum (PAS), the trophoblast inserts abnormally impairing the decidua basalis and gradually invading the entire uterine wall [25], in contrast to normal pregnancies, where extended trophoblastic infiltration ceases when it reaches the decidual spongiosa [26].

Although the least-frequent form of PAS, placenta percreta represents the most severe form, sometimes leading to serious complications, such as premature labor, heavy peripartum hemorrhage, and trophoblastic penetration beyond the uterine wall [27,28].

The placenta accreta spectrum presents several controversies, namely (i) the definition discordance regarding clinical, ultrasound and histopathological criteria [3,29]; (ii) the inclusion in the same spectrum of diseases of both categories, placental adhesion abnormalities and placental abnormal invasion, each of them with different histopathological aspects; and (iii) potential confusion between a retained placenta and an abnormally adherent placenta [3,30,31].

In this regard, placental retention cannot be included in the same category as PAS because this condition manifests when the placenta remains inside the uterus after delivery or evacuation, even if it separates from the uterine wall [3,20]. Although a retained placenta or product of conception is rare, it has an increasing prevalence due to the growing number of cesarean sections and abnormal placentation [4,5,6,20,32,33,34]. Both the retention of placental fragments or product of conception and placenta percreta have a risk of postpartum bleeding, with a frequency of 3–5% after vaginal delivery [1].

Given that most similar cases are resolved by conservative treatment, the corresponding case reports do not present the histopathological examination for diagnostic certification [1,3,4,5,6,35].

Arteriovenous uterine malformation (AVM) is a female genital pathology with specific descriptive imaging elements due to macroscopic abnormal communications between arteries and veins, without the intervention of the capillary network [17]. Ultrasonography may highlight these characteristics [36].

At the same time, diagnostic ultrasounds can detect limited and painful mobility of the organs due to adhesions formation [37,38], as it was in our case, where the reduced movement of the left ovary raised the suspicion of postoperative adhesions.

In our case, for the initial ultrasound, we used the transabdominal route, through which an area of 31/32/28 mm was pointed out, with a heterogeneous echostructure, in the anterior uterine wall, concordant with similarly reported data in the literature [39,40].

At this stage, the differential diagnosis may take into account the following uterine pathologies: (i) Uterine fibroids, which usually have a capsule, absent in our case; (ii) adenomyosis, which modifies the echostructure by altering the endo-myometrial line, with intrauterine characteristic ectopic foci near the endometrium, or in our case, the abnormal structure involving uterine serosa; (iii) isthmocele, where the hernia of the cesarean scar has a triangular appearance, characteristic of the uterine isthmic region (Table 1) [1]. Moreover, the preoperative imaging examination can also take into account a differential diagnosis of leiomyosarcoma or degenerating fibroids [41].

The case management could be guided by following the peak systolic velocity (PSV) of the flow inside the malformation. When PSV is higher than 0.83 m/s, the cases respond better to permanent surgical treatment, while those with PSV under 0.4 m/s may respond to conservative treatment [42].

If β-HGC dynamics are also taken into account, the differential diagnosis of arteriovenous malformation includes pathologies with increased vascularization where it can be assumed to be a neoangiogenesis process, possible in a gestational context [43]. Thus, the relatively common forms of gestational trophoblastic disease (GTD), invasive mole and choriocarcinoma may present vascular arteriovenous abnormalities, but in these cases, β-HGC values are higher than the ones seen in our patient [44,45]. A particular situation is represented by the placental site trophoblastic tumor (PSTT), which represents a variant of the gestational trophoblastic disease with an arteriovenous shunt, but with lower β-HGC values [46,47]. Not least, retained products of conception (RPC) may show increased vascularization predominantly of the systolic arterial type and mainly with endometrial involvement [35,39]. In these situations, β-HGC values are moderately increased (Table 2) [48].

In our case, the arteriovenous malformation was acquired, as long as the patient did not have any menstrual disorder as a marker of abnormal vascularization. Moreover, uterine curettage and previous cesarean sections also represented conditions for its occurrence, in accordance with those reported in the literature [10,11].

Historically, the first case of uterine AVM was reported in 1926 [39,49]. The acquired form of uterine AVM occurs more frequently in women of fertile age [39].

Although the mechanism is still unknown, several studies mention, along with myomectomy, uterine dilation and curettage (D&C), cesarian section, the existence of pregnancy, along with β-HGC variations, which may play a role in the development of an otherwise latent AVM [17,35,39]. Moreover, these surgical procedures, along with GTD or endometrial carcinoma, can cause uterine trauma in the presence of an arteriovenous malformation, which makes curettage—the specific treatment in the case of uterine bleeding—contraindicated, in the context of an AVM [17,35]. This could possibly justify the alteration of the patient’s condition through persistent severe hemorrhage after the performed diagnostic curettage.

Arteriovenous malformation is usually identified in multiparous patients with abundant and intermittent bleeding, usually due to high vascular flow across the lesion because of the different pressure degrees between the arterial and venous systems [35]. Literature data report that approximately 50% of patients with acquired AVM need transfusions, a situation encountered in our case as well [17,50].

Moreover, a history of recurrent miscarriage indicates an increased risk of AVM, as patients may remain asymptomatic [35].

Histological examination of the hysterectomy specimen revealed the irregular and dilated blood vessels in the myometrium, which confirm the uterine AVM, corresponding to similar features reported in the literature [41]. Sometimes, in this context, one can note an atypical aspect of bland endothelial cells of the malformed arteries and venules with abnormal vascular dilation and blunt changes in their media thickness, such as papillary endothelial hyperplasia in the lumen of thrombosed vessels, but without presenting atypia, mitosis, or necrosis [39,41].

Another distinct aspect of our report was the histopathological confirmation of the retained product of conception, revealed by the presence of fibrotic placental villi in the uterine wall, considering that the microscopic certification of persistent trophoblast or placental tissue by detecting chorionic villi represents the characteristic feature that certifies this condition [9].

The difficult cases, which rule out malignant or other benign tumors, can be supplementarily highlighted by special stains or immunohistochemical techniques [41]. In this context, the concern of a malignant condition also requires extensive sampling of the surgical uterine specimen [41].

The particularities of the presented case consist of the association of the abnormal placentation, in the form of a retained placenta percreta, with an acquired uterine AVM leading to a radical surgical approach. Radical surgery made the histopathological examination possible, as, in general, the diagnosis and treatment of AVM do not require extensive surgery that can provide microscopical certification of injuries.

To the best of our knowledge, there are only a few reported cases in the literature with similar diagnostic associations as in our presentation, as well as with a radical therapeutic decision due to the occurrence of massive bleeding and suspicion of gestational trophoblastic disease [20,21,22,23,24,51].

Conservative treatment methods [17,24,35,52] were also considered in our case, but the medical context of our patient led to the choice of radical surgical therapy, with a hysterectomy being preferred when the patient does not want to preserve fertility [4,6,19].

Operative hysteroscopy [52] was also taken into account in our case, with this method being described in the literature as a good variant fertility-sparing technique in the context of AVM [52]. When there is significant hemorrhage, which can mask the visualization of the cavity and cauterization of the malformation, added to other diagnostic suspicions (placental site trophoblastic tumor or placenta accreta), the treatment should be radical to avoid a possible life-threatening risk [52].

Moreover, hysterectomy is the therapy of choice in the context of complications due to abnormal placentation, retained product of conception and arteriovenous malformation, all of which lead to acute and severe bleeding, events that can endanger the patient’s life [3,6,7,13,18,20,25,33,42,44]. A similar context was in our case, in which the surgical therapeutic decision was also justified by the suspicion of gestational trophoblastic disease and the lack of the patient’s fertility desire. Therefore, as the patient declined additional gestational planning, a total hysterectomy, bilateral salpingectomy, left oophorectomy, with right external iliac lymph node sampling and extended adhesiolysis were performed. Our multidisciplinary team decided on adnexectomy due to extended adhesiolysis followed by intraoperative bleeding, which made it difficult to preserve the left ovary. Because of the suspected throphoblastic tumor following MRI examination and biopsy results, lymph node sampling was performed. Our therapeutic strategy was in accordance with data reported in the literature [53,54].

An increasingly used therapeutic approach is endovascular transcatheter uterine artery embolization (TCUE), which, since its appearance in the late 1980s, has proven to be a safe and less-invasive method, especially for patients who want to preserve their fertility [17,35]. Thus, in the presence of a uterine AVM, the unilateral or bilateral variant of TCUE must be taken into consideration [35].

However, the literature data show that the second follow-up embolization has a higher success rate compared to the first embolization [35]. Moreover, published studies have not led to clear treatment guidelines for cases in which the initial embolization did not succeed, in order to compare the effectiveness of a second embolization with that of hysterectomy or medical therapy, in the case of persistent hemorrhage [35].

Another study presents the therapeutic results of 62 patients who received TCUE associated with pelvic angiography, with a failure rate of 29%, where the persistent uterine hemorrhage was resolved at different intervals by a secondary embolization, hysterectomy or bilateral laparoscopic ligation of the uterine artery. Although beneficial in treating arteriovenous fistula and bleeding cessation, the embolization method remains controversial in maintaining the patient’s fertility [39].

Given the aspects presented above, as well as the reported increased maternal morbidity, in the case of placenta accreta treated with TCUE [55], together with the specific medical context of our patient, the multidisciplinary team decided to perform a hysterectomy instead of TCUE.

A special technique reported in one study was the use of ureteral stents, in the case of a cesarean hysterectomy for the placenta accreta, to avoid involuntary urinary tract injury, but results showed that the method does not reduce these risks [56].

## 4. Conclusions

Retained placenta percreta associated with an acquired arteriovenous malformation is a rare but sometimes life-threatening condition. Besides other benign or malignant diseases, the gynecologist should include in the differential diagnosis the uterine AVM in the presence of a massive and persistent unexplained uterine hemorrhage, associated with significant β-HGC variations and specific Doppler imaging in a premenopausal woman.

Total hysterectomy is taken into consideration when the acquired AVM is associated with abnormal placentation and the patient does not want to maintain fertility. In this rare context, a histopathological examination may provide a definite diagnosis for these conditions.

## Figures and Tables

**Table 1:** Differential diagnosis for heterogenous uterine wall echostructures.

Differential Diagnosis	Characteristic Ultrasound Features
Uterine fibroids	Capsule
Adenomyosis	Alteration of endo-myometrial line
	Intrauterine characteristic ectopic foci
	Location near endometrium
Isthmocele	Triangular appearance of cesarean scar hernia

**Table 2:** Differential diagnosis of arteriovenous malformation, according to β-HGC dynamics.

Differential Diagnosis	Characteristic Ultrasound Features	β-HGC Values
Invasive mole Choriocarcinoma	Arteriovenous abnormalities	Higher Over 50,000 mUI/mL With rapid increase
PSTT	Arteriovenous shunt	Lower Over 100 mUI/mL With variable increase
RPC	Increased systolic arterial type vascularization	Moderately increased Over 100 mUI/mL Slight decrease

## Data Availability

All data are available within the article.
